# What lives on and in the sea turtle? A literature review of sea turtle bacterial microbiota

**DOI:** 10.1186/s42523-022-00202-y

**Published:** 2022-09-08

**Authors:** Samantha G. Kuschke

**Affiliations:** 1 Department of Biomedical and Diagnostic Services, College of Veterinary Medicine, University of Tennessee, 2407 River Drive, Knoxville, TN 37996 USA; 2 Department of Biological sciences, Florida Atlantic University, 777 Glades Rd, Boca Raton, FL 33431 USA

**Keywords:** Microbiome, Microbiota, Sea turtle, Conservation, Gut, Cloacal, Nasal, Oral, Skin

## Abstract

Within the United States, all populations of sea turtles are listed as threatened or endangered under the Endangered Species Act. Identifying methods of supporting health, preventing disease, and treating disease is essential for conservation and management strategies for all species. Over the last few decades, advances in technology and high throughput sequencing have allowed a proliferation of research into core microbiota and microbiomes in humans and animals. Such investigations have proven that microbiota on and within a host can influence physiology, immunity, and development. Accordingly, a comprehensive understanding of microbiota is essential for unearthing the complex relationships within a microbiome and how those interactions and relationships can be used to promote health and prevent or treat disease. The goal of this review is to summarize the current microbiota research available across all species of sea turtles and identify any emerging trends. Methodological differences made it challenging to draw conclusions across studies, but it is apparent that each anatomical location investigated has a unique core microbiota with some potential overlap. In the future, unifying methodology across microbiota studies will allow broader conclusions to be drawn across all anatomic locations and species of sea turtles. These conclusions will then allow clinicians and conservationists to apply the research results in the field. Additionally, future efforts should include a wider range of organisms including fungi, viruses, parasites, epibiota, and archaea to unveil essential relationships among and between the organisms and host for maintenance of a healthy microbiome.

**Supplementary Information:**

The online version contains supplementary material available at 10.1186/s42523-022-00202-y.

## Introduction

Most animals are home to a complex array of microbiota, which is defined as communities of microorganisms originating from multiple different kingdoms [1, 2]. Microbiota, in combination with their spectra of activity, make up what is known as a microbiome [1, 2]. This encompasses the microbial structures, metabolites, mobile genetic elements, and relic DNA [1–3]. Within a microbiome, there is a subset of microbes referred to as the core microbiota [1, 2]. Core microbiota are a subset of the microbiota that are shared across a species in similar habitats [1, 2]. A comprehensive understanding of core microbiota is essential for unearthing the complex relationships within a microbiome such as stability, plasticity, and individual purposes or functions [1, 3].

Through the last few decades, advances in technology and high throughput sequencing have allowed a proliferation of research into core microbiota and microbiomes [3–6]. These advances have supported rapid and more affordable investigation into the microbiomes of human and nonhuman animals [3, 4, 6]. Interest in these studies is growing and stems from increasing understanding that microbiota can significantly impact host functions [7–10]. Research thus far has revealed that microbiota may influence host physiology, immunity, and development [7, 9–11]. Significantly, the microbiota most investigated across all species include those of the gastrointestinal tract and skin.

The microbiota of the gastrointestinal tract aids in metabolic homeostasis, digestion, and provides key nutrients to the host [8, 12, 13]. More surprisingly, the gastrointestinal microbiota also plays an essential role in multiple host physiologic process [8, 12, 13]. Morphogenesis of the gastrointestinal tract as well as development of secondary gut associated lymphoid tissue within the intestines partially depends on postnatal microbial colonization [8]. The gastrointestinal microbiota can also modulate the differentiation of some subsets of immune cells and production of cytokines, chemokines, and other soluble immune mediators [8, 10, 11]. Additionally, intestinal vascularization, tissue regeneration, carcinogenesis, bone homeostasis, and behavior can all be influenced by the gastrointestinal microbiota [8, 13–15].

Though somewhat less explored in animals, the skin microbiota is also a highly important network of microorganisms. The skin serves as a primary barrier and defense against the outside world [16–22]. Commensal microbiota of the skin aid in defense via production of inhibitory compounds or by competing for resources necessary for growth [16, 17]. Skin microbiota are believed to play a significant role in host health, host resistance, immune function, response to endogenous and exogenous stressors, and disease clearance [17–22]. A protective skin microbiota in amphibians has exhibited the ability to alter disease outcomes [19, 22]. Harris et al. demonstrated that the amphibian skin microbiome can decrease both morbidity and mortality associated with skin diseases caused by *Batrachochytrium dendrobatidis* (Bd) [19]. Becker et al*.* identified significant differences in skin microbiota among Panamanian golden frogs that cleared Bd infections versus those that died, suggesting that skin microbiota play an important role in clearance of Bd [23].

Investigations into the microbiota of marine animals has also become an area of interest due to their constant contact with salt water [24–27]. Some of this work includes studies in marine turtles. There are seven extant species of sea turtles, most of which are imperiled. Within the United States, all populations are listed as threatened or endangered under the Endangered Species Act [United States. The Endangered Species Act as Amended by Public Law 97–304 (the Endangered Species Act Amendments of 1982)]. They are also listed on the International Union for Conservation of Nature (IUCN) red list as vulnerable, endangered, or critically endangered (IUCN Red List). The only exception is the flatback turtle (*Natator depressus*), which is listed as data deficient. Marine turtles are often considered sentinels of the ocean due to their long-life spans and expansive migratory patterns [28]. Accordingly, large research efforts are aimed at understanding the effects of climate change on the health, physiology, and disease processes of these sentinel animals to aid conservation efforts across the world [28]. A small portion of this research has been aimed at gaining knowledge of the microbiota and microbiomes of sea turtles. The primary focus of microbiota research in sea turtles has been the microbiota contained within the gastrointestinal tract, with smaller portions of the research branching into the microbiota found in the cloaca, mouth, and nares [Additional file 1: Table S1].

The principal goals of microbiota studies in sea turtles aim to gain knowledge and understanding to improve husbandry in rehabilitation settings, improve survival outcomes in eggs, and improve treatment and prevention of disease processes [29–32]. In addition to these goals, a small subset of research has also been aimed at the identification of microorganisms that could serve as potential human pathogens, such as multidrug resistant bacteria [33–36]. The objective of this paper is to investigate the current microbiota research available from all species of sea turtles. Literature with a focus or clear relation to core microbiota of the sea turtle were the focus of this investigation. Using these essential studies, I summarize the microbiota found on and within sea turtles, identify emerging trends, and highlight areas for future focus. I have organized the review into subsections covering different anatomical locations of the sea turtle. I begin with the gastrointestinal tract and subdivide this into sample types used. I then go into mucosal surfaces, the skin and the shell.

In 2021 the International committee on Systematics of Prokaryotes changed the names of multiple phyla to align with the International Code of Nomenclature of Prokaryotes. The research presented in the review was performed prior to this change and therefore use the previously recognized names for phylum described. To maintain consistency among the literature being referenced, I use the previous phyla names (Table 1).

**Table 1:** Old and new phyla names

Old name	New name
Proteobacteria	Pseudomonadota
Firmicutes	Bacillota
Actinobacteria	Actinomycetota
Bacteroidetes	Bacteroidota
Fusobacteria	Fusobacteriota
Spirochaetes	Spirochaetota

In 2021 the International committee on Systematics of Prokaryotes changed the taxonomic names of 42 prokaryote phyla to align with the International Code of Nomenclature of Prokaryotes. Above is the list of the previously used and the new nomenclature. The old names are used for the phyla within this paper.

### Gastrointestinal tract

Accurately sampling the gastrointestinal microbiota in living animals can be difficult. Due to the challenging nature of obtaining gastrointestinal samples, I have categorized gastrointestinal microbiota research in several fashions. The first being whether the samples were obtained from live animals, or if the samples were taken following death, either natural or via euthanasia. While sampling post-mortem allows direct samples to be taken from different portions of the gastrointestinal tract, there are likely multiple factors that may have altered the microbiota prior to sampling such as illness, injury, anorexia, or dehydration [37]. Sampling from live sea turtles can also have biases depending on if the animal is wild caught, stranded, debilitated or in a rehabilitation center [31, 38]. Additionally, samples used as a proxy (i.e., cloacal, and fecal samples) to estimate the gastrointestinal microbiota in living turtles can influence the results as well [37]. Across the literature cloacal swabs and fecal samples are often used to infer the microbiota population of the gastrointestinal tract, although there are several papers that sample the colon via deep swabs through the cloaca [Additional file 1: Table S1]. The sample used and potential for contamination should always be taken into consideration when analyzing data in these studies.

#### Postmortem gastrointestinal samples

In 2018, Kittle et al. performed an investigation into the effect of the herbicide glyphosate on the microflora of green sea turtles (*Chelonia mydas*) in Hawaii [29]. Through traditional culture methods, they identified 4 taxa of bacteria from the gastrointestinal tract of 8 green turtles that required euthanasia. The taxa identified included *Proteus* sp., *Pantoea* sp., and *Shigella* sp.; all within the phylum Proteobacteria and the class Gammaproteobacteria. The fourth taxon identified was *Staphylococcus* sp. in the phylum Firmicutes. All 4 taxa proved to be sensitive to glyphosate in a dose dependent manner. It is important to note that the region of the gastrointestinal tract from which these taxa were isolated was not described and could range anywhere from the crop to the colon [29].

Also using traditional culture techniques, in 2020, McDermid et al. investigated different sections of the gastrointestinal tract from 8 Hawaiian green sea turtles that required euthanasia after mortal injury or terminal illness [39]. Samples were collected from the crop, stomach, small intestines, cecum, and large intestines. Culture and biochemical reactions were used to identify 11 taxa within the phylum Proteobacteria and 2 isolates within the phylum Firmicutes. Among the sections of the gastrointestinal tract sampled, the small intestines had the most taxa isolated at 13, followed by the stomach and cecum each with 12 taxa identified. The large intestine had the fewest number of taxa isolated at only 6. In addition to the culture samples, 2 whole gastrointestinal tracts were used to sample the cecum, large intestines, and rectum. Using high through put sequencing the most dominant phyla were identified as Firmicutes and Bacteroidetes. At the order level, Clostridiales and Bacteroidales predominated. Additionally, at the family level Clostridiaceae, Ruminococcaceae, Lachnospiraceae, Porphyromonadaceae, and Bacteroidaceae were most abundant [39].

In 2020, Ahasan et al. performed a more comprehensive investigation of the mucosa-associated bacterial communities within the gastrointestinal tract of 4 stranded green turtles that died 1 to 4 days after being taken to a rehabilitation facility [40]. The oesophagus, stomach, small intestine, and large intestine were sampled in each turtle and high throughput sequencing was used to identify the microbiota present in each section. Across the entire gastrointestinal tract, the relative abundance (RA) of the dominant phyla were Firmicutes 57.8%, Proteobacteria 21.3%, Actinobacteria 6.4%, Bacteroidetes 3.6%, and Fusobacteria 2.4%. Firmicutes, Proteobacteria, and Actinobacteria were present in all regions of the gastrointestinal tract. In the small intestines, it was noted that there was a drastically lower abundance of Firmicutes and a significantly higher abundance of Proteobacteria. There were 30 dominant families identified that belonged to 11 different classes: Clostridia, Gammaproteobacteria, Bacilli, Actinobacteria, Bacteroidia, Alphaproteobacteria, Fusobacteria, Epsilonproteobacteria, Spirochaetes, Coriobacteria, and Erysipelotrichia. The nature of this study allowed comparisons between the sections of the gastrointestinal tract to be made. Some of the significant differences noted among sections included: (i) the absence of Actinobacteria in the large intestines and the higher abundance in the oesophageal samples, (ii) Peptostreptococcaceae was significantly more abundant in the stomach and large intestines, and (iii) Clostridia was one of the most abundant classes across all regions except in the small intestines which, had a significantly lower abundance of that class. At the level of genera, a total of 459 taxa were identified across the whole intestinal tract but over 35.8% of the total sequences could not be identified. Of the operational taxonomical units (OTU) identified, 11 of them were shared across all gut regions. The phylum Firmicutes accounted for 8 of the 11 OTUs shared across the entire gastrointestinal tract. The other 3 OTUs fell within the phlya Proteobacteria, Bacteroidetes, and Fusobacteria. While this study provides a very in-depth look at the gastrointestinal microbiota of the green turtle, limitations of the study include the small sample size and the poor health status of the turtles [40].

Abdelrhman et al. found similar dominant microbiota in an investigation of loggerhead sea turtles (*Caretta caretta*) from the Tyrrhenian Sea east of Italy [41]. A portion of the study used colorectal samples from 6 recently deceased loggerheads and investigated the microbiota via high throughput sequencing. Like the findings in Ahasan et al., Firmicutes was the phylum with the highest percentage of occurrence; the percent of occurrence was reportedly higher in loggerheads at 87% [41]. Firmicutes was followed by the phyla Proteobacteria at 4.2% occurrence and Bacteroidetes at 3.4% occurrence. Furthermore, Abdelrhman et al. also found that the classes Clostridia and Bacilli dominated within Firmicutes with 43% and 42.5% occurrence respectively [41].

Across all postmortem samples, there appears to be a common thread at the phylum level. Firmicutes, Bacteroidetes, and Proteobacteria are continually identified as predominant phyla, though found at differing abundances in each individual study. However, it is important to note that with all postmortem studies, the individual turtles sampled likely had several complex physiological abnormalities present, which are apt to alter the normal gastrointestinal microbiota. Therefore, these studies may represent the change one would expect to see in the microbiota of a sick, injured, or debilitated sea turtle. Additionally, the window between the time of death and the time of sampling should also be considered when interpreting data from post-mortem samples. Extended post-mortem windows may allow for extensive amounts of bacterial overgrowth and may alter findings.

#### Fecal samples

Multiple studies have investigated the gut microbiome in sea turtles using next generation sequencing on fecal samples. Species sampled thus far include green turtles, Kemp’s ridley (*Lepidochelys kempii*) and loggerhead turtles. Campos et al. sampled 8 live captive and 11 live wild caught juvenile green turtles, while Samuelson et al. sampled 30 live incidentally captured juvenile Kemp’s ridley turtles following rehabilitation for external injuries. Both Campos et al. and Samuelson et al. used next generation sequencing and identified Bacteroidetes as the most prominent phyla and Firmicutes as the second most abundant phyla in the fecal samples. A significant increase in the abundance of Proteobacteria was noted in association with prolonged captivity [31, 42] and treatment with antibiotics [31]. Both studies postulated that that increases in Proteobacteria could be an indication of dysbiosis in sea turtles [31, 42]. In direct contrast, Bloodgood et al. reported no significant differences between juvenile green sea turtles treated with and without antibiotics [30]. In 2020, Bloodgood et al. sampled feces from 17 individual sea turtles at three time points across rehabilitation (admission, mid-rehabilitation, and recovery) and performed next generation sequencing to identify bacteria present. Dominant phyla detected at recovery were Bacteroidetes (RA 38.4%), Firmicutes (RA 31.8%), Verrucomicrobia (RA 5.45%) and Proteobacteria (RA 1.8%) [30], which aligns with the two dominant phyla identified by Campos et al. and Samuelson et al. In contrast, at admission, the most abundant phylum was Firmicutes (RA 55%), followed by Bacteroidetes (RA 11.4%) and Proteobacteria (RA 6.2%) [30]. If, as suggested by Campos et al. and Samuelson et al., an increase in Proteobacteria indicates dysbiosis, this would mean that the green turtles included in Bloodgood et al. had fecal dysbiosis at admission, and rehabilitation lead to a more normal bacterial community dominated by Bacteroidetes and Firmicutes. If this interpretation is correct, then the assumption that lengthy stays in captivity or rehabilitation centers cause dysbiosis does not hold firm for all cases, especially for turtles suffering from illness that may be secondarily altering the gut microbiota. Biagi et al. also found that time in a rehabilitation center did not impact any feature of the gut microbiota (alpha diversity, beta diversity or RA) in loggerheads [43].

While it was found that in green [42] and Kemp’s ridley [31] sea turtles Bacteroidetes are more abundant than Firmicutes, Arizza et al. found the opposite was true in loggerhead sea turtles [44]. Across 9 fecal samples sequenced from live stranded loggerhead sea turtles, Firmicutes was identified as the dominant phylum with a RA of 49.4% [44]. Bacteroidetes (RA 21.5%) was the second most abundant phylum and Proteobacteria (RA 11%) the third [44]. Two additional studies also found Firmicutes as the most dominant phylum in loggerhead sea turtles [41, 43]. The first, obtained 58 fecal samples from 29 live loggerheads stranded or captured by fishery net that were housed for rehabilitation [43]. Among these samples, the major phyla identified included Firmicutes (RA 46.5%), Fusobacteria (RA 26.5%), Bacteroidetes, and Proteobacteria [43]. The second study sampled 4 hospitalized loggerheads following stranding [41]. Firmicutes was the most abundant phylum with a RA of 66%, followed by Proteobacteria (RA 23%) and Bacteroidetes (RA 6.2%) [41]. The shift in dominance from Bacteroidetes to Firmicutes in loggerhead sea turtles in comparison to green sea turtles has been suggested to be secondary to differences in diet between the species [44]. Adult green turtles eat a primarily herbivorous diet, while loggerheads from post-hatching into adulthood are carnivorous feeders [44, 45]. Though, if diet were the driving force, you would expect the gut microbiota of the Kemp’s ridley sea turtle would more closely resemble that of the loggerhead sea turtle as their diets are more closely aligned [45].

Patterns of microbiota in fecal samples across studies and among species are more difficult to identify at lower levels of taxonomy. These difficulties arise from differences in methodology, collection time, and populations sampled. However, regardless of these challenges, a few taxonomic classes and families have been identified as abundant in multiple studies. The family identified across the most studies was Clostridiaceae [30, 41–44, 46]. Clostridiaceae has been identified as an abundant family in wild, and captive green sea turtles [29, 42, 46]. Bloodgood et al. reported that Clostridiaceae and an unclassified family from the order Clostridiales were both significantly more abundant at the time of admission to rehabilitation than at mid-rehabilitation or recovery time points [30]. Clostridiaceae has also been identified as a prominent family in loggerhead sea turtles [41, 43, 44]. Abdelrhman et al. reported that the Firmicutes phylum was dominated by the class Clostridia with the most represented genera, Clostridium XI (RA 21.3%) and *Clostridium sensustrict* (RA 14.6%), falling within the family Clostridiaceae [41]. Additionally, Biagi et al. reported a 17.8% RA of Clostridiaceae with the *Clostridium* genus having a RA of 14.8% [43]. Other commonly identified families of bacteria reported at differing RA’s across fecal studies include Bacteroidaceae, Lachnospiraceae, Ruminococcaceae and Fusobacteriaceae [29, 31, 41–44]. While the studies mentioned may not agree exactly on the relative abundance of each phylum, class, family, or genus of bacteria in the sea turtle’s feces, there is an emerging pattern suggesting that a normal core microbiota of sea turtles likely exists. Continued investigation of the fecal microbiota in healthy turtles, with a more uniform approach, would help illuminate these developing patterns.

There are multiple factors that may alter the microbiota detected in the feces of sea turtles, including but not limited to health status, diet, age, location, treatment, and debris [46–48]. Ahasan et al. investigated the effect of antibiotics versus bacteriophage therapy on the fecal microbiota of juvenile green turtles raised in captivity from emergence [46]. The dominant phyla identified across all treatment and control groups were Firmicutes, Bacteroidetes and Proteobacteria. Within these phyla, the genera *Clostridium* and *Bacteroides* were identified as the most predominant. Bacteriophage treated turtles were observed to have transient changes to their fecal microbiota with a specific decrease in the targeted microbe, while treatment with enrofloxacin resulted in a gradual decrease in fecal diversity, which did not recover immediately after discontinuing the antibiotic. Antibiotic treatment resulted in an increase in the phyla Firmicutes, and more specifically, an increase in microbes within the class Clostridia. Also observed were small decreases in the abundance of previously dominant phyla including Bacteroidetes, Proteobacteria, Verrucomicrobia, and Actinobacteria. It is important to note that this study took hatchlings immediately following emergence from the nest and raised them in a lab for 11 months [46]. Lack of exposure to the normal oceanic environment and diet during the early stages of life may have innately changed what is considered normal microbiota in the control group. Regardless of if the normal microbiota identified are equivalent to the wild counterparts, treatment with a broad-spectrum antibiotic, such as enrofloxacin, has the potential to alter the ratios of dominant bacterial phyla within the fecal microbiota. Additionally, the resulting alterations in fecal microbiota do not quickly return to normal levels following discontinuation of the antibiotic [46].

Ingestion of foreign items is a threat to sea turtles and is likely the result of indiscriminate feeding or mistaking foreign objects as food, as such there is growing concern about the effect that plastic may have after ingestion [45]. Ingested plastic debris can act as a vehicle for microbes, chemical pollutants, and toxic compounds [45, 47]. Additionally, plastic debris have the potential to cause epithelial damage to the gastrointestinal tract during passage and can result in local inflammation, which may alter the environment within the gastrointestinal tract [47]. Biagi et al. 2021 investigated the effect of plastic debris on the fecal microbiota of 45 stranded or captured loggerhead sea turtles in the Northwestern Adriatic Sea [47]. Forty-eight operational taxonomic units (OTUs) were noted to be increased in the presence of plastic debris in the feces. The most dominant species identified within this group was *Cetobacterium somerae* belonging to the phylum Fusobacteria. While this species of bacteria was previously noted to be normal fecal microbiota of loggerhead sea turtles by Biagi et al. in 2019, the relative abundance of the microbe was notably increased in the presence of plastic debris [43, 47]. Other genera that increased with an increase in plastic debris included *Fusobacterium, Vibrio, Psychrobacter, Romboutsia, Desertihabitans, Staphylococcus, Terriporobacter,* and *Gordonibacter* [47]. A number of genera were seen to decrease in abundance with an increase in plastic debris. Genera in this category included *Clostridium, Faecalicatena, Akkermansia, Rikenella, Cloacibacillus, Pseudoflavonifractor,* and *Romboutsia* [47]. Plastic debris appears to be correlated with changes to the fecal microbiota and should be kept in mind when sampling feces in wild sea turtles for gastrointestinal microbiota studies, especially in areas where plastic pollution is high [47]. Accordingly, health status, diet, age, location, medical treatment, and plastic debris should be accounted for when investigating and interpreting the fecal microbiota of sea turtles.

#### Distal colon swabs

Distal colon swabs (i.e., swabs that enter via the cloaca and are extended a minimum of 60 cm into the distal colon) are the best method we currently have for sampling the gastrointestinal microbiota in healthy living sea turtles [49]. While this method does not allow for understanding of individual sections of the gastrointestinal tract, it is likely less affected by outside factors such as water and sand [49]. Thus, it is an ideal way to identify the normal microbiota in healthy sea turtles and can aid in the interpretation of post-mortem samples collected. Comparisons between live distal colon swab samples and post-mortem gastrointestinal samples will allow researchers to identify changes in the microbiota that occurred post-mortem and those that occurred anti-mortem and may be due to significant pathology.

Using this advantageous technique on healthy adults, Scheelings et al. investigated the gut microbiota of geographically distinct populations of nesting female loggerhead and flatback sea turtles (*Natator depressus*) [49]. The loggerheads sampled included 6 from Florida, USA and 18 from Queensland, Australia. The flatbacks included 19 from Crab Island, Queensland Australia and 10 from Port Hedland, Western Australia. Across all loggerheads sampled the most abundant phylum was Proteobacteria. The other prominent phyla, identified from the US population in order of abundance included Actinobacteria, Bacteroides, and Firmicutes. In contrast, the dominant phyla identified from loggerheads in Australia included Spirochaetes, Bacteroides, and Actinobacteria. Between the two locations 37 OTUs were shared, but each location had unique OTUs not identified in the other location. Twenty-seven OTUs were unique to the US loggerheads and 61 were unique to the loggerheads sampled in Australia. The alpha diversity was noted to be significantly different between the two populations of loggerheads based on location. A similar trend of differences based on location was seen in the two flatback populations. The predominant phyla in turtles sampled from Crab Island was Firmicutes, while Proteobacteria was the most commonly identified phylum from the turtles sampled at Port Hedland. There were 28 OTUs shared between the two flatback populations, but again each location had unique OTUs. Five OTUs were identified as unique to Crab Island and 61 were unique to Port Hedland. Alpha diversity was again noted to be significantly different between the two populations. This study suggested that geographic location plays a role in shaping the core microbiota of the gastrointestinal tract. It is important to note that additional factors, such as foraging location, could not be accounted for in this study. For example, nesting females often return to the same nesting beach, but each beach serves as a nesting ground for females from numerous foraging locations [49].

In 2020, Scheelings et al. also reported microbiota data obtained from nesting females of all seven extant sea turtle species via distal colon swabs [48]. The most abundant bacterial phyla identified across all species included Proteobacteria, Bacteroidetes, Actinobacteria, and Firmicutes. Kemp’s ridley sea turtles had three phyla identified that were found in no other species: Euryarchaeota, Deferribacteres, and Cyanobacteria. There were clustering patterns of beta diversity among the leatherbacks and green turtles, which are the more ancient species. Regardless of species, Proteobacteria was the most predominant phylum identified in the colon of all sea turtles. Given this common finding among all species, Scheelings et al. suggested that if Proteobacteria is not the most abundant phylum in a nesting female it should be considered abnormal. This statement seems to be in direct contrast to those made by Campos et al. and Samuelson et al. stating that an increase in Proteobacteria is associated with dysbiosis [31, 42]. It is important to note that nesting female turtles undergo prolonged periods of anorexia [48]. This anorexia may lead to a normal shift in the gut microbiota that results in an increased abundance of Proteobacteria. Campos et al. and Samuelson et al. sampled turtles at a different life stage and physiologic stage than that of a nesting female turtle [31, 42]. At certain life stages, a shift in microbiota to Proteobacteria may be indicative of dysbiosis, while in a nesting female it may be a normal change secondary to prolonged inappetence during the nesting season.

Further investigation in healthy sea turtles at all life stages will be important for identifying the core microbiota of each species. Broad application of distal colon swabs assessed via next generation sequencing in healthy sea turtles will tremendously expand our knowledge of the sea turtle gastrointestinal microbiota. Expansion of baseline microbiota data in healthy individuals will allow definitive identification of a core gastrointestinal microbiota. Once this core is identified that knowledge can then be used and applied to husbandry, treatment, and disease prevention in sea turtles both in the wild and in captivity.

### Mucosal surfaces

Mucosal surfaces such as the cloaca, oral cavity, and nasal cavity are prime environments to support microbial communities [50]. The normal flora at these locations often serves as a primary defense against opportunistic and pathogenic bacterial colonization [50]. Mucosal surfaces are located at the interface of the host and the environment and therefore can generally be easily accessed and sampled from living animals [50]. Uniquely, mucosal surfaces are generally in constant contact with the aquatic environment that sea turtles live in [51]. Consequently, changes in mucosal microbiota may be affected by changes in the environment such as increased temperature, pH, and pollution [52, 53]. These alterations in microbiota could be detected and used as evidence that a pollutant is present and negatively impacting normal physiology of sea turtles [53]. Additionally, many sea turtle mucosal surfaces are contiguous with the gastrointestinal tract and can be used to help understand the gastrointestinal microbiota [40, 51]. More specifically, cloacal samples are often used to make inferences about gastrointestinal microbiota but are more accurately grouped as a part of external mucosal surfaces [32, 40, 41, 51]. As such, this section includes the current studies investigating the microbiota of the nasal cavity, cloaca, and the oral cavity.

#### Nasal samples

Several studies have utilized traditional culture methods combined with biochemical tests to identify the bacteria of the nasal cavity, but they are difficult to interpret as they compare the results to cloacal samples and do not clearly separate them [34, 35, 54]. In 2006, Santoro et al. used culture to isolate and identify the nasal and cloacal microbiota of nesting green turtles [54]. The gram-positive isolates were predominately composed of Staphylococcus species, which made up 73.2% of all gram-positive microorganisms. Of the gram-negative isolates, 53.1% of them belonged to the Enterobacteriaceae family, including the genera *Enterobacter* sp.*, Escherichia* sp.*, Klebsiella* sp. and *Serratia* sp.. The most identified microorganism from all samples was *Klebsiella pneumoniae*, with a 47.1% and 38.5% prevalence in nasal and cloacal samples respectively [54]. In 2008, Santoro et al. performed another culture-based study to identify the nasal and cloaca microbiota of nesting female leatherback sea turtles (*Dermochelys coriacea*) [34]. Similarly, this study reported that a predominant portion of the gram-negative isolates (60/113) fell within the Enterobacteriaceae family. Isolates included species from the genera *Enterobacter* sp.*, Escherichia* sp.*, Klebsiella* sp.*, Proteus* sp., and *Salmonella* sp. [34]. Contrary to the gram-positive profile proposed in the green sea turtle [54], 6 out of the 10 gram-positive isolates from the cloaca of leatherbacks were identified as *Enterococcus faecalis* [34]. Also isolated from the nasal cavity and cloaca of leatherbacks were *Bacillus* sp.*, Aeromonas* sp.*, Pseudomonas* sp., and *Vibrio* sp. [34]. Both *Aeromonas* sp. and *Pseudomonas* sp. were identified more frequently in the cloaca, while *Bacillu*s sp. was more prominent in the nasal cavity. Of the *Bacillus* sp. isolates, 96.3% came from the nasal samples [34]. In a culture-based investigation for antibiotic resistant Vibrio spp., Zavala-Norzagaray et al. identified microbiota from the nasopharynx and cloaca of green and Olive ridley sea turtles [35]. The identified microorganisms were again dominated by gram-negative bacteria within the Enterobacteriaceae family, including species within the genera *Citerbacter* sp.*, Escherichia* sp.*, Edwarsiella* sp.*, Morganella/Proteus* sp., and *Providencia* sp. In addition to these genera, numerous species of *Vibrio* sp. were identified. Isolates found solely in the cloaca included *Citerobacter freundii*, *E. coli*, *Morganella*/*Proteus*, *Providencia*, and *Vibrio fluvialis*. *Vibrio alginolyticus*, *Vibrio cholera*, and *Vibrio parahaemolyticus* were isolated from both the nasopharynx and the cloaca. Isolates found only in the nasopharynx included *Edwarsiella* spp., *Vibrio furnisii*, and *Vibrio* spp. [35].

Although differences in methods make it difficult to draw conclusions across studies, it is apparent that each mucosal surface has a unique microbiota with some potential overlap. These comparison studies make it increasingly evident that clearly separating each anatomic location during analysis is necessary to get the most accurate understanding of the core microbiota at each site. Additionally, unifying methodology across studies aiming to identify core microbiota will allow a broader picture to be drawn across all anatomic locations and species of sea turtles.

#### Cloacal samples

Keene et al. investigated cloacal samples from green and Olive ridley (*Lepidochelys olivacea*) sea turtles and cultured microbiota similar to those previously mentioned [55]. Cloacal samples were dominated by gram-negative bacteria in the Enterobacteriaceae family. Within this family *Serratia* sp.*, Enterobacter* sp.*, Klebsiella* sp., and *Salmonella* sp. were isolated. Gram-positive isolates commonly identified included species within the genera *Corynebacteria* sp.*, Bacillus* sp., and *Staphylococcus* sp. Nine bacteria were isolated from cloacal fluid samples during this study and were not found in sand samples. The bacteria unique to the cloaca included *Pseudomonas aeruginosa*, *Serratia plymuthica*, *Enterococcus faecalis*, *Enterobacter cloacae*, *Klebsiella* sp., *Salmonella* sp., *Bacillus* sp., *Staphylococcus epidermidis*, and *Enterobacter sakazakii* [55].

Culture based investigations of the cloaca of loggerhead sea turtles have also been performed and reveal similar microbial profiles to those found in green and Olive ridley sea turtles [33, 36]. Blasi et al. screened for gram-negative bacteria via cloacal swabs in 33 wild caught juvenile loggerhead sea turtles [36]. Of the 33 turtles sampled, 23 were classified as healthy and 10 individuals were classified as weak. Ninety enteric microbes were cultured and isolated from all 33 swabs; of them 59% of the isolates belonged to the family Enterobacteriaceae and 31% belonged to the family Shewanellaceae. Due to the nature of this study, comparisons between the healthy and weak groups of turtles were made and unique genera were identified within each group. *Hafnia, Achromobacter, Leclercia*, and *Proteus cibarius* were isolated only from turtles categorized as healthy. *Acinetobacter*, *Kluyvera intermedia*, and *Serratia* were identified only in turtles grouped as weak. It was also stated that *Klebsiella, Morganell*a, and *Providencia* were notably more prevalent in healthy turtles. Additionally, a list of core bacteria was identified across all turtles, though at varying amounts within individuals. The core microbiota included *Shewanella* sp., *Klebsiella oxytoca*, *Morganella morganii*, *Providencia rettgeri, Enterobacter* spp*., Citrobacter freundii, Pseudomonas* sp., and *Vibrio* spp. [36].

In 2020, Alduina et al. performed a broad study to identify antibiotic resistant bacteria in loggerhead sea turtles in the Mediterranean Sea [33]. The study included swabbing the organs of 8 fresh dead turtles and swabs from the cloaca, oral cavity, and skin from 14 loggerheads upon arrival for rehabilitation. The samples were cultured, and isolates were identified using a combination of biochemical enzymatic tests and sequencing. Across all samples, the most abundant organisms isolated were *Aeromonas* spp., *Citrobacter* spp., and *Enterobacter* spp.. Among the 8 cloacal samples taken, several genera within the family Enterobacteriaceae, including *Citerobacter* sp.*, Enterobacter* sp.*, Escherichia coli,* and *Klebsiella* sp., were isolated. *Pseudomonas* spp. and *Aeromonas* were also isolated from cloacal samples [33]. Across the studies utilizing culture methods to identify the microbiota within the cloaca, bacteria within the family Enterobacteriaceae appear to dominate across all three species of sea turtles investigated thus far [33, 36, 55]. Though, it should be noted that the two studies performed on loggerhead sea turtles [33, 36] were focused more on identifying antibiotic resistant bacteria. This focus likely created a bias in the culture results towards gram-negative bacteria.

In addition to the previously mentioned studies utilizing traditional culture techniques, there are also an array of studies that have used high throughput sequencing to identify the microbiota on the cloacal surface of sea turtles. Three of these studies collected cloacal samples from wild captured green sea turtles [38, 51, 56]; two of which found similar profiles with Firmicutes and Bacteroidetes as the dominant phyla [38, 51]. Price et al. investigated the cloacal microbiota in wild caught juvenile green turtles from three different habitats (pelagic, beachfront, bay) in the Northern Gulf of Mexico [51]. Eighteen cloacal swabs were obtained and Bacteroidetes and Firmicutes composed 30% of all bacteria identified from all three habitats. Firmicutes was represented almost entirely by the class Clostridia, with the family Ruminococcaceae being found solely in turtles from pelagic habitats and the family Lachnospiraceae being found only in turtles from bay habitats [51].

Similar dominant phyla were identified in 8 deep cloacal swabs from wild caught green sea turtles on the Great Barrier Reef [38]. Ahasan et al. reported Firmicutes as the most abundant phyla with a RA ranging from 60.5 to 62.6% and Bacteroidetes with a RA ranging from 27.6 to 31.9% [38]. In this same study, Ahasan et al. also performed cloacal swabs on 4 stranded green sea turtles from the Great Barrier reef. Samples from stranded green sea turtles were dominated by Proteobacteria (RA 47.6%), followed by Bacteroidetes (RA 19%), Firmicutes (RA 18.7%), and Fusobacteria (13.6%). Statistical comparisons were made between the wild caught and stranded turtles and a significant difference in bacterial diversity was identified. A higher RA of Proteobacteria was associated with stranded turtles [38]. A high abundance of Proteobacteria was also reported by Ahasan et al. in 2018 during an investigation of 4 hospitalized green turtles before and after rehabilitation [32] . At both pre-hospitalization and post-rehabilitation, Proteobacteria was the most dominant phylum. Prevalent phyla were determined via calculating cumulative abundance (CA). Phyla in order of most abundant to least from pre-hospitalization samples were Proteobacteria (CA 33.6%), Firmicutes (CA 25.5%), Bacteroides (CA 14.4%), and Fusobacteria (CA 9.1%). Cumulative abundance of each phylum post-rehabilitation was Proteobacteria 36.9%, Bacteroidetes 25.4%, Fusobacteria 16.1%, and Firmicutes 14.2% [32].

In direct contrast to the two other studies on wild caught green sea turtles [38, 51], McNally et al. 2021 found an abundance of microbial families within the Proteobacteria phylum in wild caught green sea turtles [56]. Twenty wild caught green sea turtles were caught off the coast of Florida adjacent to the St. Martins Marsh Aquatic Preserve of Crystal River. Among all 20 samples the families Neisseriaceae and Arcobacteraceae were found to have the highest percent mean abundance (MA) at 29.2% and 14.7% respectively [56]. These findings are more comparable to samples obtained from stranded and hospitalized green sea turtles [32, 38] and brings forth the question, what external factors may be affecting the cloacal microbiota?

In 2021, McNally et al. also used high throughput sequencing to investigate the cloacal microbiota of 30 wild caught Kemp’s ridley sea turtles in the same area off the coast of Florida [56]. The most abundant families identified, and their corresponding percent mean abundances were Cardiobacteriaceae 16.5%, Flavobacteriaceae 15.5%, and Neisseriaceae 10.4%, all of which fall within the phyla Proteobacteria or Bacteroidetes. Also in 2021, McNally et al. investigated the cloacal microbiota of cold stunned Kemp’s ridley sea turtles [37]. Cloacal samples were collected at intake to the New England Aquarium and again when the animal was declared clinically healthy. The determination of clinically healthy for turtles not treated with antibiotics was defined as ready for release and was based on appetite, physical examination, and transport ability. For turtles treated with antibiotics, clinical health was defined as 30 days past the termination of antibiotic treatment. The most prominent family identified at intake was Vibrionaceae with a mean abundance of 23.1%. The other three predominant families identified at intake were Arcobacteraceae, Shewanellaceae, and Rhodobacteraceae with mean abundances of 11.8%, 7.7%, and 6.7% respectively. All four of the predominant families fell within the phylum Proteobacteria [37]. In contrast, in clinically healthy Kemp’s ridley sea turtles, there was an increase in the family Flavobacteriacea (MA 17%), making it the most abundant bacterial family. The other predominant families identified in clinically healthy individuals were Vibrionaceae (MA 13.6%), Arcobacteraceae (MA 10.3%) and Rhodobacteraceae (MA 9.1%). Like in wild caught Kemp’s ridley sea turtles, the most predominant bacterial families of clinically healthy individuals all fell within the phyla Bacteroidetes or Proteobacteria [37]. Additionally, McNally et al. identified a trend of increasing Shannon diversity from intake to the time that an animal was identified as clinically healthy. Consequently, this study concluded that the microbiota of cold-stunned Kemp’s ridley sea turtles can be affected by several factors including but not limited to disease status, local environment, and antibiotics [37].

Across studies using next generation sequencing on cloacal samples, there appear to be emerging patterns of microbiota within sea turtle species, location, and health status. Further investigations into sub populations of sea turtles will continue to delineate these patterns. It should be highlighted as a note of caution that cloacal samples are often used to make inferences about the gastrointestinal microbiota of sea turtles. While this can give an idea of common enteric microorganisms found in this area, it is influenced by multiple environmental factors due to a turtle’s constant emersion in water [51]. Price et al. even suggested that the microbiota of the cloaca appeared to be more influenced by environment than by the gut [51]. Additionally, it is becoming clear that microbiota of any given site is influenced by many other factors such as age, location, and disease status [37]. For this reason, broad inferences between populations or to gut microbiota must be made with caution and should take into consideration the other factors that may have a role in the establishment of the cloacal microbiota.

#### Oral samples

Oral microbiota in sea turtles have been investigated through both culture and next generation sequencing methods. In 2020, Alduina et al. obtained 6 oral swabs from loggerheads upon arrival for rehabilitation [33]. Through culture techniques *E. coli*, *Klebsiella* spp., *Pseudomonas* spp., *Citerobacter*, and *Aeromonas* were all identified in the buccal cavity [33].

In 2021, McNally et al. investigated oral microbial communities in wild caught green and Kemp’s ridley sea turtles [56]. Within the oral cavity of green sea turtles four predominant families of bacteria were identified, with three falling under the phylum Proteobacteria, including Pasteurellaceae (MA 44.8%), Arcobacteraceae (MA 15.6%), and Campylobacteraceae (MA 9.9%). In contrast, the Kemp’s ridley turtles most predominant family Flavobacteriaceae (MA 34.8%) falls under the phylum Bacteroidetes. After Flavobacteriacea, Arcobacteraceae (MA 11.6%) was the next most abundant family followed closely by Rhodobacteraceae (MA 8.7%) [56].

Similarly, McNally et al. also found that Flavobacteriaceae had the highest mean abundance of all bacterial families when sampling the oral microbiota of cold stunned Kemp’s ridley sea turtles at intake and then again when clinically healthy [37]. Mean abundance of Flavobacteriaceae was reported as 30% at intake and 22.5% after being declared clinically healthy. There were differences in predominant families and their reported mean abundance for oral samples taken at intake versus those taken from individuals deemed clinically healthy. At intake, the other dominant families were reported to be Rhodobacteraceae (MA 13.7%), Vibrionaceae (MA 9%), and Porticocaceae (6%) [37]. In contrast, in turtles that were deemed to be clinically healthy, Rhodobacteriae had an increased mean abundance at 20.6%, followed by the presence of an unassigned Gammaproteobacteria family (MA 12.1%), and Saprospiraceae (MA 10.8%). Regardless of health status or captivity of Kemp’s ridley sea turtles, Flavobacteriaceae appears to make up an essential portion of the oral microbiota. Furthermore, the oral microbiota is persistently dominated by bacterial families falling within the phyla Bacteroidetes and Proteobacteria in all species sampled thus far [37, 56].

### Skin and shell

Investigations into the microbiota on the skin of sea turtles are lacking. Currently there is only one study reporting skin data from 2 turtles [33]. The skin is an incredibly important organ as it is an animal’s primary barrier and defense against the outside world [45]. In sea turtles, the skin is also in constant direct contact with their aquatic environment making it of great interest. In hard-shelled (cheloniid) sea turtles the epidermal-dermal unit is categorized into two groups, scutes and scales [45]. The scutes are thick stratum corneum that form plates to make up the shell [45]. The shell has 3 distinct regions, the carapace, the plastron, and the bridge [45]. The scales cover the rest of the body including the head, neck, and appendages [45]. The differing microscopic composition of the skin in these locations is important to consider when investigating the microbiota of the skin. Different regions of the shell, head, and appendages may be home to different microbiota. Additionally, the leatherback sea turtle (*Dermochelys coriacea*) has a skin composition unique from all other sea turtle species and lacks prominent scalation and heavy cornification [45]. Consequently, leatherback skin microbiota should be investigated on its own as it may be highly different from other sea turtle species.

The previously mentioned study performed by Alduina et al. in 2020 included skin swabs from 2 loggerhead sea turtles upon arrival for rehabilitation [33]. Traditional culture techniques were used and isolated *E. coli*, *Klebsiella* spp., *Pseudomonas* spp., and *Aeromonas* [33]. There is no current literature citing the use of next generation sequencing on skin samples in sea turtles. However, there are studies that investigated the skin and shell microbiota of freshwater turtles such as the red-eared slider (*Trachemys scripta*) and the Krefft’s river turtle (*Emydura macquarii krefftii*) [57, 58]. These studies suggest that there are unique microbiota located on different parts of the skin, such as the head and the shell with and without algae [57, 58]. Microbiota located on the skin, plastron, and carapace of sea turtles is an area in need of investigation. Additionally, unique consideration should be given to regions of the skin that are colonized with epibiota and algae. The concentrations of other organisms, both commensal and parasitic, could alter the core microbiota of the skin at a given location [57, 58].

### Sand, nest and eggs

Summarizing all microbiota data from sand, nests, and eggs to date is beyond the scope of this review as this review aims to summarize the microbiota found on and within the sea turtle. However, it is important to note that studies investigating the presence of microbes in the nest and on the outside of sea turtle eggs do exist but are often performed on dead eggs [59]. As such, there is a high likelihood of contamination from sand, humans, predators, and other unknown variables that make the data difficult to interpret. Additionally, unhatched eggs found after emergence are not a good representation of what would be found on the outside of a normal and healthy egg prior to hatching. With that said, an investigation of the outer microbiota of the eggs as soon as they are released from the mother would be beneficial. Currently, there are some studies that sample eggs as they fall from the cloaca, but they often focus on pathogen identification as opposed to identification of core microbiota [60–62]. There is still a large knowledge gap regarding the core microbiota of the sea turtle egg. A study using next generation sequencing, aimed at identifying the normal microbiota on the egg’s surface could provide insight as to where the core microbiota, both skin and gastrointestinal, come from. Furthermore, identifying the source of the microbiota may help labs and rehabilitation centers support a more natural development of core microbiota in hatchlings and post hatchlings.

## Conclusion

In the last few years high throughput sequencing has significantly aided the advancement in baseline knowledge of microbiota in sea turtles. Specifically, the knowledge of core microbiota within the gastrointestinal tract of sea turtles has received a significant amount of attention and advancement because of the increasing access and affordability of sequencing technology. The next step for the gastrointestinal microbiota research is to shift aims at testing hypotheses to produce meaningful and applicable clinical knowledge for sea turtles. Such knowledge could include methods of detecting and treating dysbiosis in sea turtles, development of pre- and probiotics for captive animals, determination of the least harmful but still effective antimicrobial therapy and identifying relationships between the gastrointestinal microbiota and important physiologic processes. However, while there have been great advances in baseline gastrointestinal tract microbiota data, there are still other large knowledge gaps within baseline microbiota research in sea turtles.

Knowledge of the skin microbiota is one such gap, with little to no research. Filling this gap and identifying core skin microbiota data may pave the way for development of topical skin pre- and probiotics for sea turtles. Furthermore, this knowledge could aid in the treatment of common skin issues in sea turtles such as infections, open wounds, or fibropapillomatosis. As for free ranging turtles, the skin’s constant contact with their environment and thus the contaminants within it may provide insight into what the turtles are being exposed to. Alterations in core microbiota may be associated with exposure to specific chemical pollutants or toxins in the water. Identification of such patterns will allow researchers and conservationists to detect contaminant exposure and may aid in the development of species-specific mitigation plans, conservation efforts, and policy changes to prevent future exposure. Additionally, specific alterations in skin microbiota could be detected in conjunction with changes in ocean temperatures and may provide additional insight into the effects of climate change on these threatened animals. Another knowledge gap exists regarding the microbiota found on external surfaces of healthy sea turtle eggs. Knowledge of what is found on healthy eggs could be used evaluate the effect of rising beach temperatures on otherwise healthy eggs and could potentially be a tool used to predict hatch success. Moreover, investigating ways to influence the success of a nest by manipulating the microbiota on the egg is an additional possibility.

Although methodological differences make it challenging to draw conclusions across studies, it is apparent that each anatomical location has a unique core microbiota with some potential overlap. Unifying methodology across microbiota studies will allow a broader picture to be drawn across all anatomic locations and species of sea turtles. Furthermore, such unity will aid with the application of the research, both clinically and in the development of conservation efforts. Finally, this review focused on bacterial communities found on and within sea turtles, but those are not the only types of microorganisms that can colonizes these regions. Future studies should investigate these same locations for the presence of a wider range of organisms including fungi, viruses, parasites, epibiota, and archaea. Including additional types of organisms in microbiome work can unveil relationships among the organisms and between the organisms and the host that are essential for the maintenance of a healthy microbiome.

## Supplementary Information

**Additional file 1**: **Table S1**. Publications on sea turtle microbiota. Included are the species of turtle(s) studied, the state of the animal(s), method of identification, time of sample, and sample type used.

## Data Availability

Not applicable.

## Abbreviations

IUCN: International Union for Conservation of Nature
Bd: *Batrachochytrium dendrobatidis*
RA: Relative abundance
OTU: Operational taxonomical units
CA: Cumulative abundance
MA: Mean abundance
